# Acinetobacter Prevertebral Abscess: Presenting as Dysphagia in a Diabetic Patient

**DOI:** 10.1155/2018/6051641

**Published:** 2018-09-04

**Authors:** R. Manmathan, T. Kumanan, J. A. Pradeepan

**Affiliations:** ^1^University Medical Unit, Teaching Hospital Jaffna, Jaffna, Sri Lanka; ^2^Department of Medicine, Faculty of Medicine, University of Jaffna, Jaffna, Sri Lanka

## Abstract

Acinetobacter species frequently causes nosocomial infection, particularly in patients receiving invasive ventilation at intensive care units for a prolonged period. Odynophagia is a rare, initial clinical manifestation of prevertebral abscess which subsequently develops when the abscess extends into the retropharyngeal space causing a midline bulge of the posterior pharyngeal wall. Here, we present and discuss a patient with uncontrolled diabetic mellitus who presented with severe odynophagia and dysphagia. He was diagnosed to have prevertebral abscess caused by a rarely reported bacteria, *Acinetobacter baumannii.*

## 1. Introduction

*Acinetobacter baumannii* is a rapidly emerging pathogen in the health care setting, where it causes infections that include bacteremia, pneumonia, meningitis, urinary tract infection, and wound infection. The organism's ability to survive under a wide range of environmental conditions and to persist for extended periods of time on surfaces make it a frequent cause of outbreaks of infection and an endemic, health care-associated pathogen, particularly in patients receiving invasive ventilation at intensive care units for a prolonged period.

Prevertebral abscess is one of the uncommon deep neck space infection, occupies the prevertebral space between the vertebrae bodies and prevertebral fascia, and extends from the base of the skull to the coccyx. Infections of the prevertebral space usually originate from contiguous spread of a cervical spine infection (such as discitis or vertebral osteomyelitis), by local instrumentation of the esophagus or trachea or by hematogeneous seeding. There is a predominance of Gram-positive organisms, the most common being *Staphylococcus aureus*. Less common organisms include various Gram-negative bacilli, mycobacteria, and fungi. Intravenous drug use, diabetes mellitus, immunosuppression, and alcoholism are known risk factors for the prevertebral abscess.

The diagnosis of a prevertebral space infection may be difficult to make clinically because most of the patients complain of back or neck pain with fever, and only one-thirds have neurologic deficits ranging from nerve root pain to paralysis. Computed tomography or magnetic resonance imaging is immensely helpful for differentiating a prevertebral space infection from other deep neck abscess. Complications of prevertebral space infections include spinal cord compression and mechanical instability of the spine. Since the prevertebral space extends from the base of the skull down to the coccyx and is contiguous with the psoas muscle sheath, it can be complicated with psoas abscess in a patient who has a prevertebral space infection.

## 2. Case Presentation

A 61-year-old male from northern Sri Lanka presented with high-grade fever, neck pain, odynophagia, and dysphagia for three days duration with the background of uncontrolled diabetes mellitus. His last HbA1c was 9.1% one month ago. His symptoms significantly interfered with his oral intake. He did not complain of cough, shortness of breath, headache, and ear, nose, or throat pain. He is a teetotaler and denied sexual promiscuity or substance abuse. There was no recent travel history of significance.

On examination, he was ill and febrile with a temperature of 102°F. Few enlarged (0.5–1 cm) tender lymph nodes were detected on the left anterior cervical chain with minimal neck swelling. Complete ear, nose, throat, and dental examinations were normal. Respiratory rate was 24/min. He maintained the saturation of 98% on room air, and he was hemodynamically stable. Rest of the physical examination was unremarkable.

Investigations showed neutrophilic leukocytosis (WCC 14.3 × 106/microlitre, neutrophils 90%) and elevated inflammatory markers (CRP 327 mg/dl and ESR 94 mm/1st hour), suggestive of a severe bacterial inflammation. Three sets of blood cultures were sterile after incubation. Renal, liver, and thyroid profiles were well within normal limits, and transthoracic two-dimensional echocardiogram was also normal.

**Figure 1:**
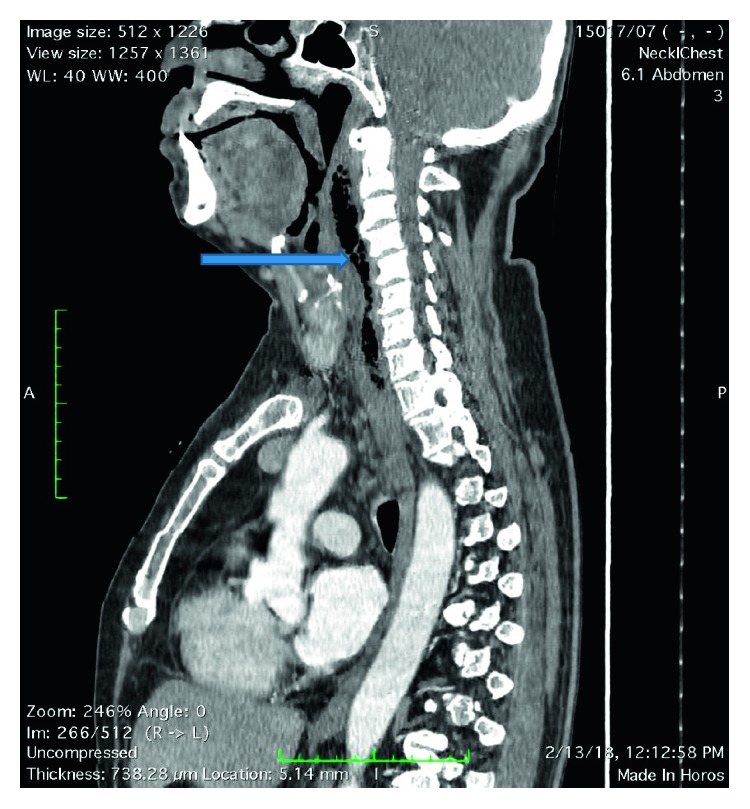
Prevertebral abscess with air extending from the C2–C7 cervical spine (blue arrow).

Fiber optic laryngoscopic examination showed fullness and inflamed pyriform fossa bilaterally with inflamed arytenoids and aryepiglottis. Contrast-enhanced CT scan of the neck confirmed a prevertebral abscess at the cervical area. Few milliliters of pus was drained before commencing intravenous broad spectrum antibiotics empirically. The aspirated pus later grew *Acinetobacter baumannii* which was sensitive to broad spectrum of antibiotics such as meropenem, imipenem, and piperacillin-tazobactam. Ultrasound scan-guided therapeutic surgical drainage of 45 ml of pus was removed via the transcervical approach by the otolaryngology team to facilitate early recovery (Figure 1).

## 3. Discussion

Prevertebral abscess is one of the uncommon deep neck space infection, occupies the prevertebral space between the vertebrae bodies and prevertebral fascia, and extends from the base of the skull to the coccyx, thus allowing organisms to spread down as far as the psoas muscle sheath [1, 2]. It accounts for less than 1% of all deep neck abscesses. It often originates from contiguous spread of a cervical spine infection (such as discitis or vertebral osteomyelitis), by local instrumentation of the trachea or esophagus or by hematogeneous seeding. The Gram-positive organism predominates, the most common being *Staphylococcus aureus*. Less common organisms include Gram-negative bacilli, mycobacteria, and fungi. Intravenous drug use, immunosuppression, HIV infection, alcoholism, substance abuse, and diabetes mellitus are known risk factors for developing deep neck space infection.

Clinical diagnosis of prevertebral abscess is often a challenge as the symptoms are inconsistent [3]. Patients may present with nonspecific neck pain, back pain, fever, or root pain due to compression or paralysis [4]. It may further complicate with spinal cord compression secondary to epidural fluid collection or psoas abscess due to the contiguous spread via the psoas muscle sheath [4].

This patient presented with high-grade fever and significant odynophagia and dysphagia which have not been commonly described with prevertebral abscess in the literature. Dysphagia and odynophagia are secondary to inflammation of the cricoarytenoid joints. Stridor and dyspnea may be the manifestations of local pressure due to pus collection. Imaging studies are the cornerstone of investigations to confirm the site and extent of deep neck abscess [1, 5]. Contrast CT or MRI scan is the gold standard test with high sensitivity and specificity. Computed tomography (CT) is the imaging modality of choice for the diagnosis of deep neck space infections. It allows critical evaluation of soft tissue and bone, localizes the infective process, defines its extent, and is an invaluable tool in planning and guiding aspiration of abscess. Magnetic resonance imaging (MRI) is not the initial modality of choice. However, when obtained, MRI is useful for assessing the extent of soft tissue involvement and for delineating vascular complications. Plain radiography is sometimes helpful for detecting retropharyngeal swelling or epiglottitis.

Management of deep neck abscess is often challenging and warrants a multidisciplinary approach. Airway maintenance, antibiotics, surgical drainage, and treatment of predisposing conditions are the key components in the management [5–9]. Transcervical or transoral approach can be carried out to drain the pus, whereas thoracotomy is indicated in mediastinal extension of abscess [2, 5, 6, 9]. Endotracheal intubation or tracheostomy should be considered before draining the abscess to protect the airway [5–7].

## 4. Conclusion

Community-acquired *Acinetobacter baumannii* infection is a rare cause of prevertebral abscess. Our patient's main clinical features such as dysphagia and odynophagia are secondary to inflamed cricoarytenoid joints.
